# Artificial Intelligence (AI) in Computer-Aided Design (CAD): How AI Is Redefining Dental CAD

**DOI:** 10.7759/cureus.107879

**Published:** 2026-04-28

**Authors:** Joseph Nassif, Wadih Nassif, Jad Sabbagh, Rita Attarian, Ghassan Moustafa

**Affiliations:** 1 Faculty of Dentistry, Lebanese University, Beirut, LBN; 2 Department of Prosthodontics, Lebanese University, Beirut, LBN; 3 Department of Prosthodontics, Saint Joseph University of Beirut, Beirut, LBN

**Keywords:** artificial intelligence in dentistry, artificial intelligence in prosthodontics, computer-aided design, digital dentistry, generative adversarial network (gan)

## Abstract

Artificial intelligence (AI) is becoming an increasingly important component of modern dentistry, particularly with the expansion of digital workflows and computer-aided design (CAD) technologies. This article explores the role of AI in dentistry, with a specific focus on its integration into digital dentistry and restorative CAD design. The objective is to clarify how AI technologies can support clinicians and dental technicians in improving accuracy, efficiency, and consistency in dental practice. The article introduces the fundamental concepts of AI, machine learning (ML), and deep learning (DL) to provide a foundation for understanding their clinical applications. It also reviews the implementation of AI across several dental specialties, including restorative dentistry, prosthodontics, implantology, orthodontics, endodontics, periodontology, oral and maxillofacial radiology, oral pathology, forensic dentistry, and digital dentistry. Particular attention is given to AI-integrated CAD systems used in fixed prosthetic design. The article further discusses current limitations of AI in dentistry, including issues related to data quality, ethical considerations, transparency, and the continuing need for clinician oversight. AI is presented as a supportive tool that can enhance digital workflows and assist dental professionals in delivering more predictable restorative outcomes while complementing clinical expertise and professional judgment.

## Introduction and background

Artificial intelligence (AI) refers to the creation of systems capable of performing tasks that normally require human cognitive abilities, such as learning, reasoning, decision-making, and language understanding [1,2]. While public interest in AI accelerated with the recent introduction of large language models, the integration of these technologies into healthcare has followed a longer evolution from early rule-based systems to modern machine learning (ML) and deep learning (DL) applications [1,3,4]. These branches differ in methodology but share a common goal of improving data interpretation and clinical efficiency across multiple medical and dental disciplines [2,3,5,6].

In the dental field, AI technologies have historically been applied to assist with radiographic interpretation, caries detection, endodontic evaluation, and the management of electronic records [3,5,7]. However, as the field moves toward a more integrated digital ecosystem, there is an increasing shift from purely diagnostic tools toward generative and predictive systems [1,6]. In restorative and prosthodontic practice, AI has become a cornerstone of modern computer-aided design and manufacturing (CAD/CAM) workflows by improving design precision, predicting prosthesis performance, and reducing fabrication time while maintaining high clinical accuracy [5,8,9]. This evolution is particularly evident in the use of specialized architectures, such as convolutional neural networks (CNNs) for automated segmentation and generative adversarial networks (GANs) for the autonomous design of complex tooth morphologies [10,11].

Despite these advances, several challenges remain. Successful clinical implementation depends on the availability of high-quality 3D datasets, rigorous validation of design accuracy, standardized evaluation methods, transparent reporting, and appropriate clinician training to ensure reliable and ethical integration in dental practice [3,7,9].

Therefore, the aim of this scoping narrative review is to clarify the role of AI specifically within digital dentistry and CAD for restorative and prosthodontic workflows.

## Review

This study employs a scoping narrative review methodology, a design selected to effectively synthesize the rapidly evolving evidence regarding AI in CAD. This approach facilitates a thematic integration of technical engineering concepts, such as GAN architectures, with clinical dental outcomes in restorative and prosthodontic workflows. The primary research question focused on how AI methodologies, specifically generative and convolutional architectures, optimize the accuracy and efficiency of dental CAD workflows. Electronic searches were performed across PubMed (Medical Literature Analysis and Retrieval System Online (MEDLINE)), Scopus, and Excerpta Medica database (Embase) using a combination of Boolean operators and keywords including “artificial intelligence in dentistry,” “artificial intelligence in prosthodontics,” “computer-aided design,” “digital dentistry,” and “generative adversarial network (GAN).” The search was limited to peer-reviewed, English-language studies published between 2021 and 2026. To ensure strict alignment with the research objective, a multi-dimensional screening process was applied; studies were included only if they utilized a defined AI methodology (e.g., GANs, CNNs, or ML), were applied specifically to digital dental design or CAD-based restorative workflows, and provided clinical or technological relevance to treatment planning and manufacturing. Exclusion criteria involved studies lacking methodological detail, purely theoretical papers without clinical application, or AI applications unrelated to CAD, such as 2D diagnostics or administrative software. The initial search results were screened by two independent reviewers to minimize selection bias, with any discrepancies resolved through consensus. Following the application of these criteria, a total of 31 articles were selected for final inclusion. Due to the high degree of methodological heterogeneity regarding AI architectures and dental outcome measures, a quantitative meta-analysis was not performed. Instead, a narrative synthesis was conducted, following principles similar to the PRISMA guidelines for transparency, to categorize the impact of AI on morphological accuracy and workflow efficiency (Table 1).

**Table 1:** Qualitative review methodology: databases, keywords, and selection criteria MEDLINE: Medical Literature Analysis and Retrieval System Online; Embase: Excerpta Medica database; GAN: generative adversarial network; CAD: computer-aided design; CNNs: convolutional neural networks; RMSE: root mean square error

Parameter	Details
Review type	Scoping narrative review
Search period	January 2021 – December 2026
Databases	PubMed (MEDLINE), Scopus, Embase
Search terms	“artificial intelligence,” “digital dentistry,” “computer-aided design,” “generative adversarial network (GAN),” “prosthodontics”
Inclusion dimension 1 (technology)	Studies utilizing specific AI architectures (GANs, CNNs, machine learning, or deep learning).
Inclusion dimension 2 (domain)	Focus on digital design workflows, automated tooth morphology, margin detection, or CAD-based restorative planning.
Inclusion dimension 3 (outcome)	Studies reporting on technical accuracy (e.g., RMSE, marginal fit) or workflow efficiency (e.g., design time reduction).
Exclusion criteria	1. AI used only for 2D diagnostics (e.g., caries/bone loss detection) without CAD integration; 2. Non-peer-reviewed sources or opinion pieces; 3. Studies lacking technical methodology for AI implementation.
Review process	Double-blind screening by two independent reviewers; conflict resolution via consensus.
Final selection	31 articles
Synthesis method	Narrative Synthesis (Justified by the high heterogeneity of AI models and dental metrics preventing meta-analysis).

Description of different AI models

Strong AI and Weak AI

AI refers to forms of non-human intelligence and is generally classified into two main categories: weak AI and strong AI [10]. Weak AI, also called narrow AI, is developed to perform specific, well-defined tasks within limited domains. The vast majority of current AI applications belong to this group. Examples include reinforcement learning systems, such as natural language processing tools like Google Translate and automated chat assistants, as well as computer vision systems used for facial recognition and image analysis [10]. In the context of dentistry, weak AI underpins many of today’s clinical tools, such as software for radiograph interpretation, caries detection, and orthodontic landmark identification, where the algorithms are designed for one targeted purpose rather than general reasoning. Strong AI, in contrast, represents a theoretical level of intelligence comparable to human cognition, characterized by self-awareness, understanding, and the ability to generalize learning across multiple contexts [10]. The goal of strong AI is to develop systems capable of independent decision-making across different domains, though research in this area remains largely experimental due to significant ethical and safety considerations, and no practical strong AI currently exists.

Within weak AI, two important subcategories are ML and expert systems. ML includes several learning strategies: supervised, unsupervised, and semi-supervised approaches, each differing in how data are used during training [10]. In supervised learning, the system is trained on labeled datasets, meaning that each example includes known outcomes. This “guided” process allows the algorithm to learn relationships between clinical inputs and diagnostic or predictive outputs, such as identifying dental caries or predicting treatment needs from radiographic data [10]. Common supervised techniques include methods such as k-nearest neighbors, logistic regression, and support vector machines [10]. Unsupervised learning, on the other hand, identifies hidden patterns within unlabeled datasets without prior guidance [10]. In dentistry, this can be used to group patients with similar clinical profiles or discover underlying patterns in oral health data. Semi-supervised learning combines aspects of both methods by using a smaller set of labeled data alongside a larger volume of unlabeled information, which is useful when expert annotation of dental images is limited [10]. A newer direction, known as weakly supervised learning, aims to minimize the cost and effort of labeling data. For example, in dental radiograph analysis, models can be trained using only image-level labels, indicating the presence or absence of a condition without needing precise boundary markings or detailed annotations [10]. These flexible learning strategies expand the potential for applying AI in dental diagnostics, education, and research [10].

Expert-Based System and ML

Within weak AI, two primary approaches are commonly recognized: expert-based systems and ML. While expert systems rely on predefined clinical rules, ML is generally categorized into supervised, unsupervised, and semi-supervised learning depending on the data processing methodology [10]. In supervised learning, algorithms are developed using labeled datasets where each input is paired with a known outcome, a method frequently applied in diagnostic support and restorative design workflows [10]. Conversely, unsupervised learning works with unlabeled data to independently identify underlying patterns or group similar cases without prior instruction, which is valuable for analyzing correlations within large dental datasets [10]. Semi-supervised learning bridges these strategies by utilizing a small amount of labeled data alongside a larger unlabeled set, a crucial efficiency in dental research where manual annotation is resource-intensive [10]. Furthermore, weakly supervised learning has emerged as a cost-effective alternative that reduces dependence on precise boundary markings, enabling models to train on general annotations to optimize clinical workflows and digital design [10]. By integrating these varied ML architectures, dental CAD systems can achieve higher levels of automation and accuracy.

DL and Neural Networks

**Figure 1:**
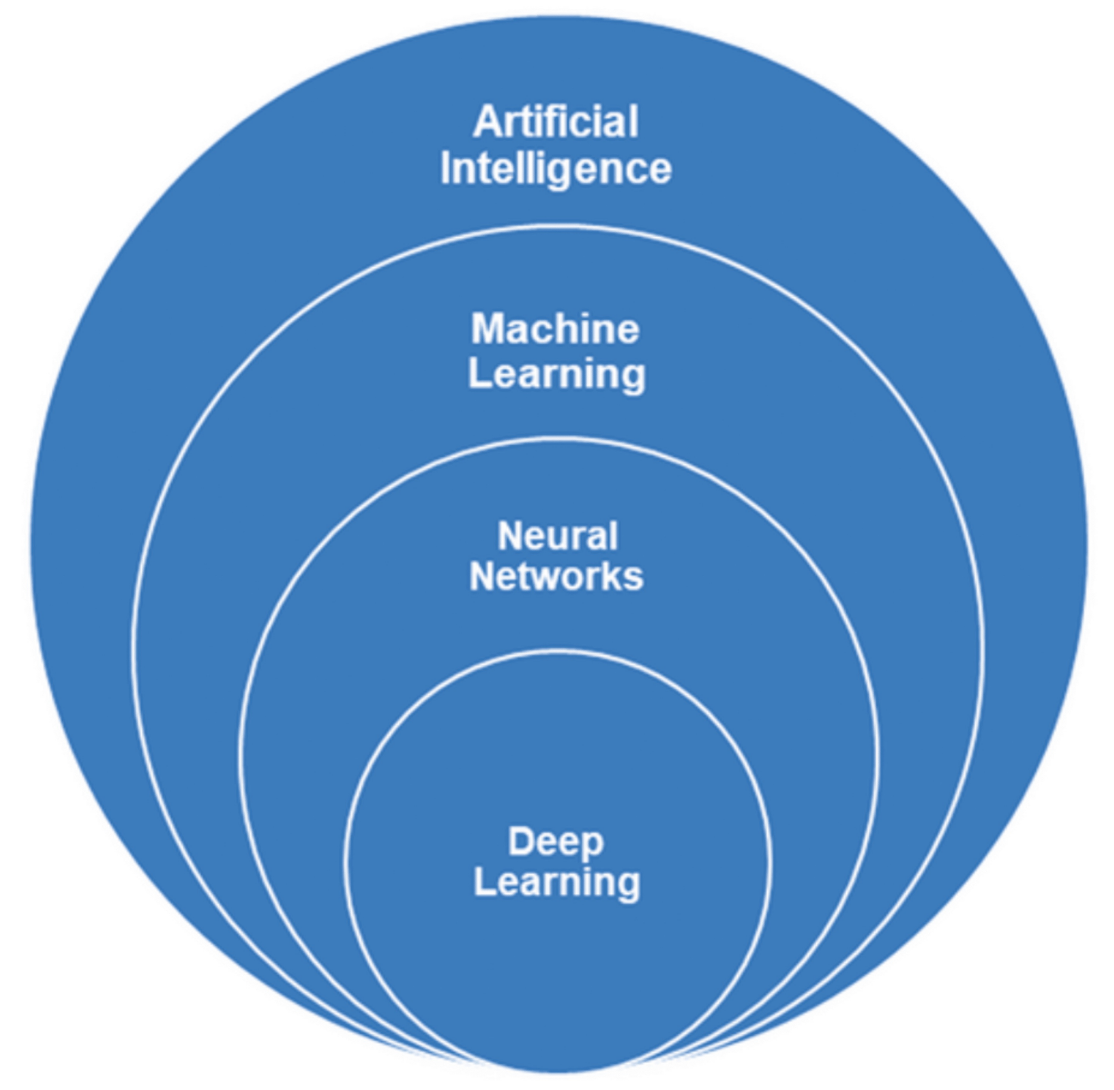
The hierarchical taxonomy of artificial Intelligence methodologies Reproduced from Tabatabaian et al. [2], J Esthet Restor Dent. 2023;35:842–859, under CC BY license.

DL, one of the most influential areas within AI, is considered a specialized branch of ML (Figure 1). It can operate under both supervised and unsupervised learning frameworks. The term “deep” refers to the multiple processing layers that make up artificial neural networks (ANNs), where data pass through several stages of analysis and transformation. These layers, typically consisting of an input layer, several hidden layers, and an output layer, allow the system to process complex information and recognize intricate patterns without explicit human instruction. A central strength of DL lies in its ability to automatically extract meaningful features from large and varied datasets, eliminating much of the need for manual data processing or feature selection. This distinguishes DL from earlier expert systems, which relied heavily on human-defined rules and input [5]. While expert systems typically require smaller, well-structured datasets, DL models achieve higher performance when trained on extensive data collections that capture clinical diversity and variability. Neural networks, inspired by biological neural structures, form the foundation of DL. Common network types include ANNs, CNNs, and GANs, each offering unique strengths in data interpretation and image analysis. Beyond theoretical design, DL has made substantial contributions to healthcare. It has been applied to identify disease patterns in medical images, predict clinical outcomes, and simulate biological or pharmacological interactions [5]. In dentistry, research in DL has expanded rapidly across diagnostic, preventive, and therapeutic domains [5]. Applications include automated caries detection from radiographs, assessment of endodontic case complexity, cephalometric landmark identification for orthodontic analysis, and classification of dental implant systems [5]. Because these approaches rely on recognizing statistical patterns within complex data, DL's multi-layered architecture provides superior performance in managing multimodal information such as radiographic images, proteomic data, and clinical records [5].

Artificial Neural Networks

**Figure 2:**
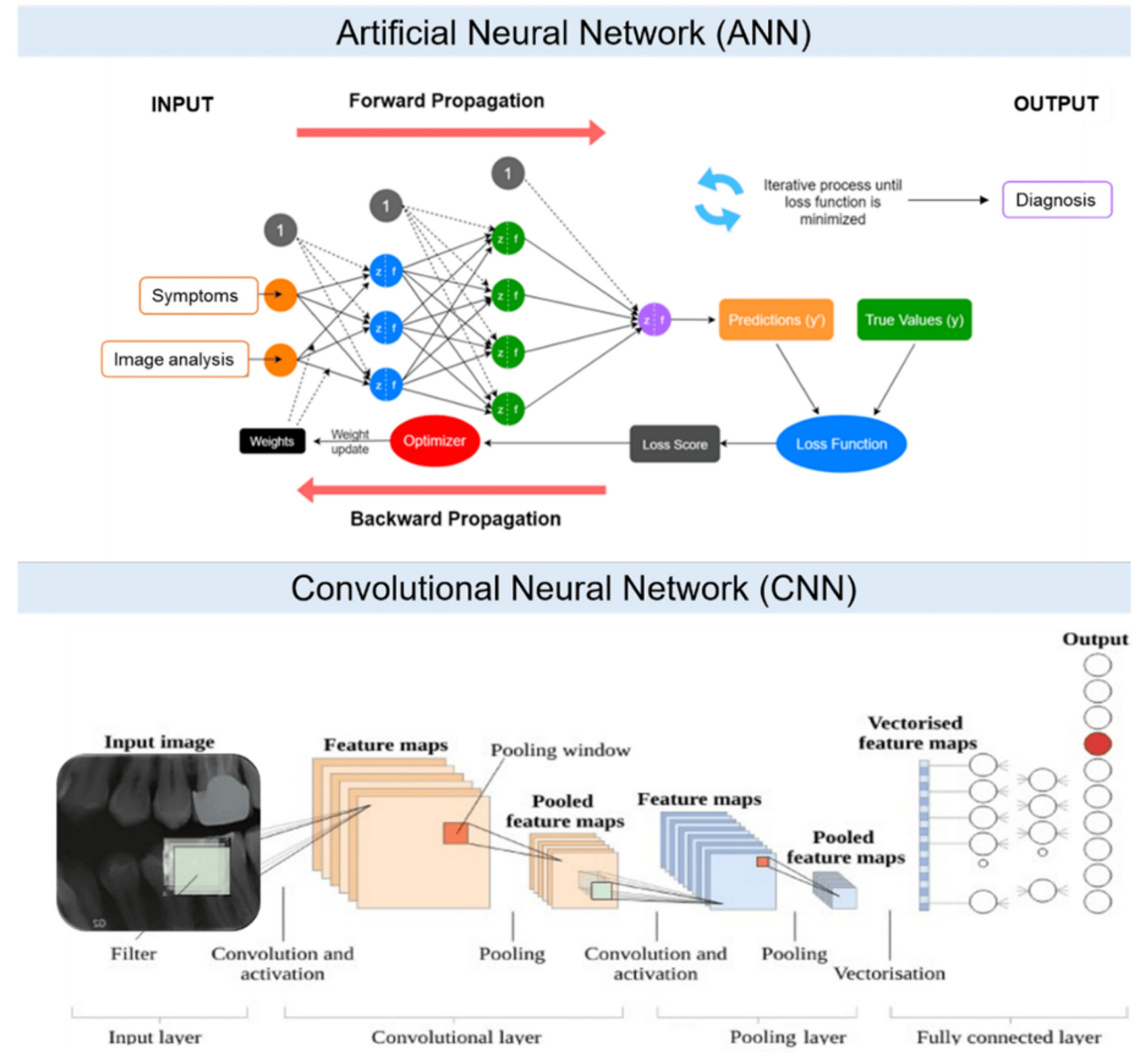
Artificial neural networks (ANNs) and convolutional neural networks (CNNs) presentations. Reproduced from Ghaffari et al. [3], Dentistry Review. 2024;1:100081, under CC BY license.

ANNs represent the fundamental structure of DL and consist of at least three layers: input, hidden, and output (Figure 2). The data flow exclusively in the forward direction, with input neurons extracting features and transmitting them to subsequent hidden layers. Each hidden layer processes the information it receives by applying weights and transformations before passing it along. This layer-by-layer processing allows ANNs to progressively capture more complex data features, ultimately producing summarized results in the output layer [10,11].

Convolutional Neural Networks

CNNs are a specialized form of DL designed primarily for image recognition and generation tasks. Unlike ANNs, CNNs incorporate convolutional layers, pooling layers, and fully connected layers within their hidden architecture. Convolutional layers apply kernels to input images, creating feature maps by systematically folding the image and reducing complexity through weight sharing. Pooling layers, which typically follow convolutional layers, further reduce the dimensionality of feature maps, enhancing efficiency in feature extraction. Finally, fully connected layers transform the 2D feature maps into a 1D vector, linking them to category nodes for classification [10]. Through this layered structure, CNNs achieve higher efficiency and accuracy in image recognition compared with ANNs.

Generative Adversarial Networks

GANs, introduced by Goodfellow et al. in 2014, represent an important advancement in DL [10,12]. As an unsupervised learning model, GANs are capable of identifying patterns within existing data and generating new data that closely resembles the original. A GAN is composed of two interconnected neural networks: a generator, which produces synthetic data, and a discriminator, which evaluates whether the generated data is authentic or artificial. Through this adversarial process, both networks continuously refine their performance, resulting in highly realistic data generation. Since their introduction, GANs have been widely applied in areas such as image-to-image translation and the creation of lifelike synthetic images of objects, scenes, and human faces [10,12]. Wu et al. further expanded this framework with the development of 3D-GANs, which integrate conventional GAN principles with volumetric convolutional networks. This innovation allows for the generation of 3D objects directly from 3D data or even from 2D images, greatly enhancing possibilities for 3D data processing beyond traditional image synthesis. In dentistry, the clinical relevance of DL models such as GANs is especially evident in prosthodontics, a field that integrates both scientific precision and artistic design in the restoration of oral structures [5]. AI integration has improved the digital design and fabrication of dental crowns and prostheses, which are essential for restoring function and aesthetics in compromised teeth [5]. DL techniques have enabled automation in crown design, manufacturing, and fitting processes, contributing to more accurate, durable, and individualized restorations. Although the field continues to advance rapidly, variations in task selection, computational methods, and performance reporting remain, prompting recent scoping reviews to address these gaps and guide future applications [5]. Beyond prosthodontics, the scope of DL continues to expand. One significant innovation is multimodal DL fusion (MDLF), which integrates data from different medical sources such as radiographs, clinical images, and patient histories to enhance diagnostic accuracy and improve disease detection [6]. Another promising development is neuromorphic computing, which merges computational models with neuroscience principles to simulate brain function. This approach holds potential for applications in drug discovery and the management of neurological disorders [6]. Additionally, speech-based analytics are emerging as valuable clinical tools. By examining vocal features such as tone, rhythm, and language patterns, AI systems can identify subtle neurological changes related to cognition, memory, and communication, offering new possibilities for early detection of neurodegenerative conditions [6]. On a broader scale, AI has also contributed to public health monitoring. For example, digital health QR codes were implemented globally during the COVID-19 pandemic to assess infection risk and track individual health status through mobile technology [6]. These diverse applications highlight how DL is evolving toward integrated, practical solutions that enhance both individualized care and population-level health management.

AI in dentistry

Advantages and Disadvantages of AI in Dentistry

AI has brought notable advantages to dentistry by streamlining routine tasks, improving diagnostic accuracy, and enabling more personalized treatment planning. AI systems can quickly analyze radiographs, detect caries or bone loss, and assist in designing prostheses with a level of precision that supports better patient outcomes. These technologies also save time, reduce human error, and enhance efficiency across clinical workflows. AI can perform several simple tasks in the dental clinic with greater precision, less staffing, and fewer errors than human counterparts, from booking and coordinating regular appointments to assisting with clinical diagnosis and treatment planning [13]. On the other hand, there are important limitations and disadvantages to consider. AI models often rely on large, well-annotated datasets, which are not always available in dentistry. This creates risks of bias, especially if the data do not represent diverse patient populations. Moreover, AI decision-making can lack transparency, making it challenging for clinicians to fully trust or interpret the results. Cost and integration issues also remain barriers for widespread adoption, particularly in smaller practices. Overall, while AI offers transformative benefits, careful oversight, validation, and clinician involvement are essential to ensure its safe and effective use in dentistry [1,10].

Applications of AI Across Dental Specialties

In operative dentistry and cariology, AI enhances caries detection and clinical decision-making, addressing limitations of conventional visual and radiographic diagnostic methods that may miss early or proximal lesions [10]. ML and DL models trained on intraoral images and radiographs can identify suspicious regions and assist treatment decisions with high diagnostic performance [2,10]. CNNs such as ResNet-152 have demonstrated an accuracy of 95.21% and sensitivity of 98.85% while also supporting restorative workflows including margin detection, radiographic tooth identification, and CAD/CAM-based treatment planning [2,14,8,9,10,15].

In periodontics, neural network models enable the detection of periodontal bone loss and classification of disease severity from radiographic images with sensitivity above 85% and accuracy exceeding 80% [1]. Predictive models integrating behavioral and systemic factors help estimate disease progression and tooth loss risk [1,4,10]. ML analyses have also explored links between periodontal disease and systemic conditions such as hypertension, obesity, arthritis, and sleep disorders [1,3,7].

In endodontics, DL systems improve detection of apical radiolucencies and periapical lesions with diagnostic performance comparable to or exceeding clinician interpretation [1,3,4,9]. Automated root canal segmentation and identification of complex canal morphologies, such as C-shaped canals, enhance treatment planning and procedural precision [1,3,7,16]. Neural network-assisted apex locators and CNN-based tools further improve working length determination and evaluation of obturation quality [1,3,6].

In orthodontics and pediatric dentistry, ML models assist in identifying dental anomalies, plaque accumulation, dentition stages, and caries risk while supporting behavioral management and patient education [1,9,17]. CNN-based systems improve cephalometric landmark detection and malocclusion diagnosis with performance comparable to human examiners [1,4,7,10]. Digital workflows integrating intraoral scanning, 3D modeling, and customized appliance fabrication further enhance orthodontic treatment planning and efficiency [4,7,10].

In oral and maxillofacial pathology and implantology, DL models analyze clinical, radiographic, and histopathological images to detect potentially malignant disorders and oral cancers with diagnostic accuracy comparable to experienced specialists [4,10]. Predictive algorithms assist surgical planning by estimating extraction difficulty, guiding orthognathic procedures, and identifying nerve pathways to minimize complications [1,7]. Integration of cone-beam CT (CBCT) imaging, intraoral scans, and CAD/CAM workflows enables personalized implant planning, bone quality assessment, and improved prosthetic design [2,6,9,3,7,11].

AI in digital dentistry and prosthodontics

Prosthodontics, a discipline that unites science and artistry to restore oral function, comfort, and aesthetics, has been profoundly transformed by AI, marking a shift from traditional manual methods toward data-driven precision [5]. AI supports a broad range of clinical and laboratory tasks, from detecting fractures, lesions, caries, and tooth wear to assisting in tooth shade selection and smile design. DL models applied to radiographic and CT imaging have demonstrated improved diagnostic accuracy and consistency [1]. Within restorative workflows, AI systems are capable of predicting the longevity of CAD/CAM restorations, assessing debonding risks, and generating individualized crown morphologies using two- and three-dimensional GANs, achieving greater precision than conventional digital tooth libraries [4,10].

In removable prosthodontics, AI assists in arch classification, denture base and occlusal scheme design, and prediction of post-insertion facial appearance, supported by models such as CNN-based detection on panoramic radiographs [4,6]. AI technologies have further enhanced critical clinical steps such as crown finish line detection and margin identification, where CNN and hybrid models outperform traditional CAD software by improving marginal accuracy and reducing microleakage [2,5]. Shade matching, a process historically influenced by subjective perception, has been optimized through neural network and fuzzy logic-based approaches that minimize observer variability, though limited dataset diversity continues to constrain universal applicability [2,5,9].

Fully digital workflows integrating AI with CAD/CAM and 3D or 4D printing systems have streamlined the fabrication of crowns, bridges, and implant-supported restorations. By combining intraoral scans with ML algorithms, these systems can design prostheses tailored to individual anatomical and aesthetic profiles, increasing efficiency and personalization [3,11,15]. AI is used during the scanning process to automatically remove excess soft tissues and material [18]. Building denture teeth in edentulous patients that meet both functional and esthetic requirements is never simple for dental professionals. ML in CAD/CAM software may restore sound inter-maxillary connections by placing the teeth correctly [19]. In implant prosthodontics, AI contributes to diagnostic accuracy, anatomical landmark segmentation, and implant stability prediction, thereby improving pre-surgical planning and long-term outcomes [6,11].

Beyond clinical applications, AI has expanded into dental education and commercial platforms. AI-driven diagnostic systems enhance radiographic interpretation in daily practice, while AI-based evaluation platforms offer standardized feedback and objective assessment in dental training environments [6,7]. Collectively, these innovations demonstrate how AI is redefining prosthodontics and digital dentistry by enhancing efficiency, precision, and patient-specific care. Nonetheless, challenges remain concerning the anatomical accuracy of digital reconstructions, variability in color calibration, and reproducibility of shade selection, emphasizing the need for continued research and refinement [1-11,15].

Limitations of Conventional CAD and the Integration of AI-Driven Solutions

CAD systems have revolutionized restorative dentistry by enabling efficient digital workflows, yet they remain limited by their dependence on manual input and fixed tooth morphology libraries, which fail to capture patient-specific anatomy [20,21]. The design stage often requires substantial technician intervention to refine occlusal morphology and marginal adaptation, leading to variability in outcomes and extended working time during both laboratory and clinical stages [22]. Even with advanced CAD-CAM integration, inconsistencies in occlusal alignment and the need for repeated adjustments are common, as the preloaded tooth templates are not tailored to individual occlusal schemes or arch relationships [20,23]. Moreover, critical processes such as finish line detection and margin definition still rely on the operator’s experience, introducing subjective variability that can compromise the marginal integrity and longevity of restorations [24].

AI offers a data-driven solution to these limitations by learning morphological and functional patterns directly from patient data. DL models, including CNNs and GANs, have demonstrated strong potential in automating the design of crowns and dental prostheses with minimal human input while maintaining or even improving accuracy [20,21]. These AI systems can analyze spatial relationships between adjacent and antagonist teeth to generate highly individualized prosthetic designs that emulate natural occlusal morphology and fit more accurately within the dental arch [22,25]. Furthermore, AI-assisted finish line detection systems, such as hybrid CNN-CAD models, have achieved superior precision compared to traditional semi-automated software, significantly reducing operator dependency [24]. Overall, the integration of AI into CAD workflows provides a transformative improvement in design efficiency, morphological fidelity, and consistency, overcoming key limitations of conventional CAD-based restorative design [20,22-24]. For AI to be successfully integrated into dental practices, clinician training is vital. Dentists and technicians must be properly trained to use AI-driven CAD tools to ensure effective and safe use in clinical settings [11].

Networks used (GANs or Deep Convolutional GAN (DC-GAN) for design, CNN for identification)

In digital dentistry, DL networks have played a pivotal role in improving automation, precision, and design efficiency. Among these, GANs, including their variants such as 3D GAN and DC-GAN, have demonstrated exceptional capability in reproducing detailed tooth morphology and occlusal features [20]. These networks function through two competing models, a generator that produces a 3D image and a discriminator that evaluates its realism, refining the output through iterative learning [25]. Systems such as Dentbird Crown (Imagoworks Inc., Seoul, Korea) and Automate (3Shape A/S, Copenhagen, Denmark) have incorporated these GAN-based architectures to autonomously generate single-molar prostheses and full-contour crowns [20,25].

Validation parameters, such as Hausdorff distance, are quantitative measures used to compare the similarity between two 3D surfaces: for example, an AI-designed crown and the original natural tooth or technician-designed model. It calculates the maximum distance between points on the two surfaces, showing how far one model deviates from the other. A smaller Hausdorff distance means the AI design is closer in shape to the reference model, indicating higher morphological accuracy. The Hausdorff threshold refers to the maximum deviation value considered clinically acceptable.

Intersection-over-union (IoU) is a performance metric used to evaluate how well two 3D objects or regions overlap, for example, how well the AI-designed tooth overlaps with the original natural tooth. A perfect match gives an IoU of 1.0 (or 100%), meaning both shapes fully coincide. In feasibility testing, 3D GAN models demonstrated strong performance, achieving a mean Hausdorff distance between 0.44 mm and 0.75 mm and an IoU of 60%, confirming that AI-generated crowns closely matched natural tooth morphology [20]. In comparison with technician-based CAD workflows, GAN-generated crowns showed similar or superior morphology and occlusal contour alignment while reducing design time by more than 60% [23]. Enhanced GAN frameworks such as StyleGAN and FusionNet further improved occlusal surface precision and internal fit consistency, minimizing manual design correction [23].

For tooth identification, segmentation, and margin detection, CNNs have become the core architecture of AI-driven dental systems [24]. CNN models, such as CenterNet-based preparation detectors and UneXt encoder-decoder networks, enable automatic identification of prepared abutments and accurate delineation of crown finish lines [24]. In the Dentbird system, CNN modules achieved 97% accuracy in finish line recognition and maintained Hausdorff distance thresholds of ≤0.366 mm for desktop scans and ≤0.566 mm for intraoral scans [24]. These metrics significantly outperformed rule-based CAD platforms such as 3Shape, exocad, and Medit, which showed higher error variability and user dependency [24].

Collectively, the integration of GANs for crown morphology generation and CNNs for anatomical identification represents a major advancement in AI-based restorative dentistry. These networks work synergistically to improve morphological fidelity, internal adaptation, and marginal precision while drastically reducing technician workload and human variability [20,23-25]. As a result, commercial AI systems such as Dentbird Crown and 3Shape Automate have set a new benchmark for digital dental design by achieving faster, more consistent, and clinically reproducible outcomes compared with conventional CAD workflows [20,23-25].

Time Efficiency

One of the major advantages of AI in digital dentistry is its capacity to significantly reduce design time without compromising accuracy. Traditional CAD systems, while efficient compared with manual methods, remain dependent on the operator’s proficiency and require extensive adjustment during design and optimization [26]. In contrast, AI-integrated CAD software has demonstrated superior time efficiency by automating most stages of the workflow, from margin detection to occlusal morphology generation [23]. In comparative analyses, GAN-based AI systems such as Dentbird Crown completed crown design processes in an average of 97-100 minutes, while the conventional 3Shape Dental System required approximately 397-516 minutes for the same tasks, representing a reduction of nearly 75%-80% in design time [26].

Additionally, GAN architectures like StyleGAN and FusionNet streamline complex occlusal and internal surface reconstructions, removing the need for multiple manual corrections and significantly shortening the overall workflow [23]. Automated margin detection through CNN-based modules, such as those used in Dentbird, further improves efficiency by eliminating manual identification steps; these algorithms complete finish line detection within seconds, maintaining accuracy thresholds within clinically acceptable ranges [24]. Furthermore, fully automated systems, including 3Shape Automate, eliminate technician-dependent variations, standardizing design speed regardless of operator skill or experience [22,25]. Overall, the integration of AI within CAD environments has transformed the time efficiency of prosthesis fabrication, allowing clinicians and technicians to achieve accurate, individualized designs with minimal intervention. This reduction in manual workload not only increases productivity in dental laboratories but also facilitates faster clinical turnaround, enhancing patient satisfaction and workflow consistency across restorative dentistry [22-26].

Accuracy

Morphological accuracy and performance: Several studies have evaluated morphological accuracy by directly comparing AI-generated restorations with crowns designed either by conventional CAD systems or by experienced dental technicians. In the study by Chau, Hsung, McGrath, Pow, and Lam, the researchers assessed whether a 3D GAN could accurately reconstruct a missing first molar by comparing each AI-generated tooth with its corresponding natural tooth [20]. Chau et al. quantified morphological similarity using the mean Hausdorff distance, which ranged between 0.44 and 0.75 mm, and the IoU, which achieved a value of 0.60, indicating that six out of 10 reconstructions successfully matched the original tooth morphology. By directly comparing AI-generated crowns to natural anatomy, Chau et al. demonstrated that GAN-based morphology synthesis can approximate real tooth features with clinically acceptable precision [20].

A more clinically oriented comparison was performed by Cho, Ahn, and Park, who evaluated two DL systems (Automate and Dentbird) in relation to crowns designed by experienced technicians using conventional CAD workflows [25]. Cho et al. examined occlusal and axial surface morphology across all three design sources, reporting mean root mean square deviations (RMSDs) of approximately 231.7 µm for Dentbird, 266.3 µm for Automate, and roughly 245-260 µm for technician-designed crowns. In addition, Cho et al. demonstrated through color deviation mapping that AI-generated crowns maintained occlusal morphology within ±50 µm across a larger percentage of the surface compared with conventional CAD crowns, highlighting the consistency and functional accuracy of DL-generated morphology [25].

In another comparative investigation, Cho et al. analyzed how GAN-generated crown morphology behaved during the design and optimization process compared with conventional CAD systems [23]. Cho and colleagues reported that the initial RMS deviation of GAN-generated crowns was 54.1 ± 25.5 µm, while the conventional CAD designs deviated by 241.3 ± 88.9 µm prior to refinement. This substantial difference demonstrated that GAN-based initial morphologies required far fewer manual modifications and inherently captured accurate occlusal and axial contours before final optimization. Cho et al. emphasized that this stability in early-stage morphology was a significant advantage of AI-generated designs over traditional CAD outputs [23].

Based on Nagata et al., AI-equipped CAD enables the fabrication of uniform, high-accuracy crowns regardless of the operator's experience level (Table 2) [26].

**Table 2:** Comparison between AI-equipped CAD and conventional CAD CAD: computer-aided design Adapted from Nagata et al. [26], J Adv Prosthodont. 2025;17:1–10.

Parameter	AI-equipped CAD (Dentbird)	Conventional CAD (3Shape Dental System)
Occlusal surface accuracy	Achieved a highly precise misfit of 25.7±13μm	Conventional CAD resulted in a significantly larger misfit of 275.5±116.8μm
Buccopalatal uniformity	Showed no significant differences in accuracy between the buccal and palatal surfaces.	Conventional CAD showed significantly larger misfits on the functional palatal side.
Proximal contact	Resulted in better contact strength (70% frequency in the ideal floss group).	Conventional CAD had a lower ideal contact frequency (40%) and more loose contacts.
Skill independence	Minimized errors caused by the individual technique or skill of the dental technician.	Conventional CAD accuracy was highly dependent on the craftsman's years of experience and skill.

Table 2 reveals a clinically significant disparity in occlusal surface accuracy between AI-equipped and conventional CAD systems. The AI-integrated system achieved a highly precise misfit of 25.7 ± 13 µm, a value well within the clinically acceptable threshold for passive fit. Conversely, the conventional CAD workflow resulted in a significantly larger misfit of 275.5 ± 116.8 µm. From a clinical perspective, gaps exceeding 100-120 µm are often associated with increased risks of cement dissolution, microleakage, and the subsequent development of secondary caries. By maintaining such precise occlusal adaptation, AI-driven systems not only improve the longevity of the restoration but also minimize the need for invasive chair-side occlusal adjustments, thereby preserving the structural integrity of the prosthesis and supporting long-term periodontal health [23,26].

Together, these studies progress from anatomical reconstruction accuracy [20] to reproducibility of clinically relevant morphology [25], to design stability prior to optimization [23]. Collectively, the findings show that AI-based systems, particularly those incorporating 3D GAN and hybrid GAN-CNN frameworks, consistently produce crown morphologies that closely replicate natural tooth anatomy, require fewer adjustments, and often achieve equal or superior accuracy compared with traditional CAD or technician-based designs [20,23,25]. Although AI-integrated CAD systems generally demonstrated equal or superior morphological performance compared with conventional CAD, some studies reported comparable outcomes between AI-generated and technician-designed crowns. This indicates that conventional CAD workflows, when guided by experienced technicians, can still achieve high morphological accuracy; however, this requires increased manual refinement and longer design times [23,25].

Marginal line identification: Accurate marginal line identification is essential for achieving proper crown adaptation and long-term restoration success. Traditional CAD workflows rely heavily on the clinician or technician to manually outline the finish line, introducing operator-dependent variability that can affect marginal integrity. In the study by Cho, Ahn, and Park, the authors compared crowns designed using DL systems (Automate and Dentbird) with crowns created by experienced technicians, demonstrating that manual or semi-automated margin identification within conventional CAD systems remains susceptible to inconsistency, particularly in areas with complex curvature or reduced visibility [25]. Cho et al. showed that deviations in marginal line placement occurred more frequently in technician-designed and conventional CAD workflows, emphasizing the need for greater automation in this critical step [25].

A more focused evaluation of marginal line detection was performed by Choi, Ahn, and Park, who developed and tested a DL-CAD hybrid method for automated finish line extraction using CNN models such as CenterNet and UneXt [24]. Choi et al. compared their hybrid AI method against three established CAD systems: 3Shape, exocad, and Medit, while using both desktop and intraoral scans. Their findings demonstrated that the AI-based approach consistently outperformed the CAD systems, especially in posterior teeth and concave marginal areas, which are traditionally challenging for manual and semi-automated methods. Specifically, the Dentbird hybrid system achieved a Hausdorff distance threshold of 0.366 mm for desktop scans and 0.566 mm for intraoral scans, values that were significantly lower and clinically more acceptable than those achieved by the comparison software [24]. Choi et al. further demonstrated that the hybrid CNN-CAD system produced fewer outliers and greater repeatability than technician-guided CAD workflows, indicating superior robustness in margin identification [24].

**Figure 3:**
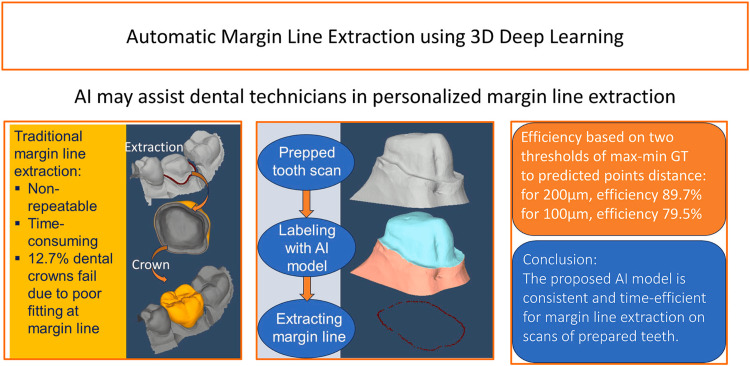
Difference between traditional margin line extraction and automatic margin line extraction using 3D deep learning. Reprinted from Computers in Biology and Medicine, volume 196, Alsheghri A, Zhang Y, Keren J, Cheriet F, Guibault F, Automatic margin line extraction using 3D deep learning on digital surface models of prepared teeth for crown generation, 110960, Copyright (2025), with permission from Elsevier [28].

By learning the geometric features that distinguish enamel, dentin, and gingival boundaries, the CNN models were able to identify the finish line continuously and accurately, even in regions where visual contrast or anatomical complexity typically undermines manual precision (Figure 3). These results strongly support the clinical value of DL-assisted margin detection, as AI systems reduce operator dependency and enhance the predictability of marginal fit [24,25]. Collectively, the work of Cho et al. and Choi et al. demonstrates that AI-driven marginal line identification not only matches but often exceeds the accuracy of conventional CAD systems and technician-dependent workflows [24,25]. Their findings highlight the potential for DL algorithms to standardize one of the most technique-sensitive steps in crown design, thereby improving the overall precision and longevity of digitally fabricated restorations. The Automate program exhibited an acceptability score comparable with that of dental laboratory technicians in finish line detection and restoration design, as well as significantly lower deviation than the Dentbird program [27,28].

Although conventional CAD systems can achieve clinically acceptable marginal line identification when operated by experienced clinicians or technicians, none of the reviewed studies reported superior performance compared with AI-integrated CAD systems. Instead, AI-based approaches demonstrated greater consistency and accuracy, particularly in anatomically complex or posterior regions (Table 3) [24,25,28].

**Table 3:** Comparison of the accuracy for finish line extraction between AI-based CAD (hybrid) and conventional CAD. CAD: computer-aided design Adapted from Choi et al. [24], J Prosthet Dent. 2024.

Parameter	AI-based CAD (hybrid method)	Conventional CAD
Clinical acceptability	Only AI software (Dentbird) met clinical thresholds for both intraoral and desktop scans.	Conventional software (3Shape, exocad, MEDIT) only met thresholds for desktop scans, failing on intraoral scans.
Consistency (SD)	Lower standard deviation indicates more consistent accuracy across various tooth preparations.	Semiautomatic conventional methods produced more outliers and inconsistent results depending on the user.
Complex convexity	Demonstrated robust detection in complex regions where concave and convex surfaces coexist.	Conventional software relies on predefined convexity, making accurate identification of complex lines impossible.
Intraoral scan accuracy	Achieved the smallest mean Hausdorff and Chamfer distances for posterior teeth.	Significantly outperformed ($P < .05$) conventional tools for concave regions typically found in intraoral scans.

Marginal fit, contact point, and occlusion: Marginal fit, proximal contact quality, and occlusal accuracy are critical determinants of the clinical success and longevity of fixed dental prostheses. To facilitate a structured comparison of the current evidence, Table 4 provides a summary of performance metrics across key studies evaluating AI-driven systems versus traditional restorative workflows.

**Table 4:** Comparative analysis of marginal fit, internal adaptation, and occlusal accuracy between AI-integrated and conventional CAD restorative workflows. RMS: root mean square deviation; GAN: generative adversarial network; CNN: convolutional neural network; CAD: computer-aided design

Study	AI Method/System	Comparator	Primary Metrics Evaluated	Key Outcome
Nagata et al. [26]	AI-equipped CAD	Conventional CAD	Internal fit (RMS)	AI achieved superior precision (25.7 µm) vs. conventional CAD (275.5 µm).
Cho et al. (2024) [22]	Dentbird, Automate	Technician	Internal gap, occlusal contacts	AI systems showed similar functional accuracy to technicians.
Cho et al. (2023) [23]	GAN-based (StyleGAN)	Conventional CAD	Internal fit (RMS), time efficiency	GAN-based systems showed lower RMS deviation (55.4 µm vs 85.6 µm).
Choi et al. (2024) [24]	CNN-hybrid method	3Shape, exocad, Medit	Finish line extraction	AI consistently outperformed CAD software in accuracy and repeatability.
Chau et al. (2024) [20]	3D GAN	Natural tooth	Morphology similarity (Hausdorff)	GAN-based synthesis achieved clinically acceptable structural similarity.

As summarized in Table 4, the heterogeneity in reported metrics and methodologies across these studies underscores the necessity for standardized reporting in future AI dental research. Despite this methodological variance, the findings demonstrate a consistent trend: AI-driven systems frequently achieve superior marginal fit and more predictable occlusal morphology compared to conventional CAD or technician-based approaches. This comparative overview contextualizes the more detailed functional analyses provided in the following studies.

In the comparative study by Cho, Ahn, and Park, the authors evaluated the internal fit and occlusal contact performance of crowns generated using two DL systems (Automate and Dentbird) alongside technician-designed crowns produced through conventional CAD workflows [25]. Cho et al. measured internal gap thickness using RMSD analysis and found that the Dentbird system achieved a mean internal gap of 59.4 ± 12.1 µm, outperforming both the technician-designed crowns (65.4 ± 10.3 µm) and Automate (83.1 ± 13.1 µm) [25]. These findings indicated that DL-based design methods were capable of generating restorations with improved internal adaptation and greater uniformity across samples. In the same study, Cho et al. also analyzed occlusal and proximal contact points to determine functional accuracy. Dentbird and Automate produced approximately 4.3 ± 1.7 to 4.5 ± 1.7 occlusal contacts per crown, closely resembling the 5.5 ± 1.5 contacts recorded for technician-designed crowns [25]. Furthermore, AI-generated crowns exhibited more even contact distribution and fewer heavy contacts, reducing the likelihood of premature occlusal interference and the need for chairside adjustment [25]. Mesial and distal proximal contact forces were also evaluated, and the DL models showed fewer instances of excessive contact intensity compared with conventional CAD designs, indicating improved anatomical and functional contour reproduction [25]. Complementing these findings, Nagata et al. performed an in-depth comparison of internal fit accuracy between GAN-generated crowns and conventional CAD designs [23]. The authors reported that GAN-based crowns achieved an RMS internal fit deviation of 55.4 ± 17.1 µm, whereas conventional CAD crowns demonstrated significantly higher deviations of 85.6 ± 29.6 µm [23]. Nagata et al. also highlighted that AI-generated crowns displayed more uniform adaptation along axial and occlusal surfaces, suggesting that the GAN models were able to capture internal geometry and seating behavior more effectively than traditional library-based CAD systems [23]. This improved adaptation reduces microgap formation, cement washout risk, and long-term biological complications. Together, the findings of Cho et al. and Nagata et al. demonstrate that AI-driven crown design systems can consistently achieve superior marginal fit, more predictable proximal contacts, and improved occlusal morphology compared with traditional CAD or technician-based workflows. By reducing internal discrepancies and minimizing high-contact areas, these AI systems enhance both the functional performance and clinical efficiency of fixed restorations, supporting their growing integration into digital dentistry [23,25]. The crowns designed using DL were found to be comparable to those designed by technicians in terms of occlusal scheme, internal fit, tooth morphology, and proximal contacts [8]. While acceptable marginal fit and occlusal contacts can be achieved using conventional CAD and technician-guided design, no evidence was found indicating superior performance compared with AI-assisted systems. AI-based workflows were instead associated with improved consistency and a reduced need for manual adjustment, particularly in internal fit and contact point accuracy [23,25].

Discussion

The integration of AI into digital dentistry represents a significant advancement in the precision, efficiency, and predictability of restorative workflows. Traditional CAD systems, while well-established, remain limited by operator dependency, fixed tooth libraries, and the need for extensive manual refinement, particularly during the design of occlusal morphology, marginal lines, and internal surfaces [20,24,25]. These limitations frequently lead to inconsistencies in morphological fidelity, marginal adaptation, and occlusal contacts, contributing to increased laboratory effort, longer chairside adjustment times, and potential biological complications. The emergence of DL models, especially GAN and CNN-based systems, directly addresses these issues by automating the most technique-sensitive steps of crown design and analysis [20,24,25]. Across multiple studies, AI has demonstrated the capacity to interpret patient-specific morphology and generate restorations that closely replicate natural occlusal and axial surface contours. GAN-based models were shown to reconstruct missing molars with mean Hausdorff distances ranging between 0.44 and 0.75 mm, indicating high structural similarity to natural teeth [20]. When evaluated against technician-designed crowns, AI systems such as Dentbird and Automate produced comparable RMSD and, in several cases, maintained occlusal morphology within narrower tolerance ranges, affirming their capacity to replicate clinically relevant anatomical details [25]. Furthermore, StyleGAN-based systems demonstrated enhanced morphological stability, requiring fewer adjustments during optimization and maintaining superior accuracy prior to final refinement [23]. This level of predictability is essential in dentistry, where minute deviations in morphology can influence occlusion, gingival health, and long-term restoration performance.

Marginal line identification, historically one of the most operator-dependent and error-prone steps in digital workflows, has also benefited substantially from AI integration. CNN-based models developed by Choi et al. demonstrated improved precision and repeatability in finish line extraction, achieving clinically acceptable Hausdorff thresholds and outperforming conventional CAD systems such as 3Shape, exocad, and Medit, especially in posterior or anatomically complex regions [24]. Accurate margin detection directly contributes to improved marginal fit, reducing microgap formation, cement dissolution, and long-term biological risk. Together with enhanced morphological precision, AI-driven margin identification significantly strengthens the reliability of the entire CAD-CAM restorative process [24,25].

Internal fit and occlusal contact quality represent additional domains where AI exhibits substantial advantages. Studies comparing Dentbird, Automate, and technician-designed crowns revealed that AI systems not only achieved tighter and more consistent internal gaps, often below 60 µm, but also produced balanced occlusal contacts comparable to those generated by skilled technicians [25]. These findings were further supported by Nagata et al., who demonstrated that GAN-based crowns maintained significantly lower RMS deviation values than conventional CAD systems, indicating better internal adaptation and improved seating behavior [23]. This has critical clinical implications: restorations requiring fewer adjustments reduce chairside time, patient discomfort, and long-term mechanical stress while increasing procedural efficiency.

Time efficiency is another domain in which AI provides a decisive advantage. DL systems automate tasks that previously demanded significant manual input, such as margin tracing, occlusal surface refinement, and internal fit correction. Comparative data show that AI-assisted design can reduce total working time by over 60%-75% compared with conventional CAD workflows, substantially improving productivity in both clinical and laboratory environments [22,23]. Importantly, these time savings do not compromise accuracy; instead, they reflect the ability of AI models to generate more anatomically faithful initial designs, requiring fewer iterative modifications [23,25].

Taken together, the evidence across these studies demonstrates that AI integration in digital dentistry yields improvements in morphological accuracy, marginal precision, internal fit, occlusal harmony, and workflow efficiency. AI not only matches but frequently exceeds the performance of conventional CAD systems, while reducing operator dependency and minimizing variability across cases. As digital dentistry continues to evolve, AI-driven restorative design represents a promising pathway toward enhanced standardization, reduced clinical workload, and improved patient outcomes [20,22-25].

Future Perspective

The future of AI in dentistry is characterized by continuous integration of data-driven decision making, increasingly autonomous digital workflows, and the progressive shift toward personalized, predictive, and precision-based patient care. As demonstrated across existing applications, AI is already capable of supporting diagnosis, treatment planning, image interpretation, and prosthetic design, but its long-term potential extends far beyond current boundaries [1,3,9]. Ongoing advancements in ML, particularly DL models such as CNNs and GANs, will drive future systems toward greater autonomy, accuracy, and clinical reliability [10,15]. In this context, digital dentistry remains the field where these developments will have the most immediate and measurable impact, especially in restorative dentistry, prosthodontics, and CAD/CAM-based workflows.

One of the most significant future directions is the evolution of AI-driven design automation. Current studies already demonstrate that GAN-based systems can reconstruct tooth morphology with clinically acceptable accuracy and consistency, often surpassing conventional CAD software and reducing the need for manual refinement [20,23,25]. As datasets expand to include diverse anatomical presentations, worn dentition, edentulous cases, and multi-unit prostheses, future models will be capable of generating full-arch restorations, occlusal schemes, and even complete digital wax-ups tailored to individual functional patterns [4,6]. Most existing algorithms rely on supervised learning, but the gradual transition toward semi-supervised and weakly supervised learning will reduce annotation requirements and allow models to learn from much larger, more heterogeneous clinical datasets [1,5]. This will make AI tools more robust, less prone to bias, and better suited for real-world clinical variability.

Future AI systems will also improve functional integration, enabling restorations that adapt not only to static morphology but also to the patient’s dynamic jaw movement. Robotics-based articulators, already capable of reproducing mandibular kinematics, are expected to integrate with AI-driven occlusal analysis to design restorations that account for functional pathways, excursive movements, and parafunctional habits [11,15]. Combining AI-generated designs with real-time motion tracking and virtual articulators may allow fully automated functional occlusion design, reducing postoperative adjustments and improving long-term stability [23,25].

Additionally, AI could support predictive modeling by analyzing long-term clinical data to estimate wear patterns, fracture risk, or prosthesis survival, thereby guiding material selection and design features based on individualized prognosis [9]. Another major future direction lies in expanding AI-assisted diagnostic and planning tools. Current applications in radiograph analysis, CBCT interpretation, and caries detection will evolve toward comprehensive decision-support systems capable of integrating clinical records, imaging, periodontal assessments, and biometric data to support multi-factorial treatment planning [3,7,10]. These tools may help clinicians detect early disease patterns, identify risk factors, and select the most appropriate treatment approach based on predictive outcomes.

In implant dentistry, AI-enhanced planning software will increasingly incorporate bone density mapping, anatomical risk assessment, and prosthetically driven implant positioning, while robotics may enable precise autonomous or semi-autonomous surgical execution [6,15]. As robotic technology becomes more accessible, the future clinical workflow may involve AI-generated treatment plans executed with robotic precision, improving predictability and reducing operator variability.

Digital dentistry will continue to benefit from enhanced AI-based segmentation, margin detection, and image processing. Studies have shown that CNN-driven segmentation systems outperform conventional CAD software in detecting finish lines and anatomical boundaries, especially in posterior regions or areas of limited visibility [24]. Future algorithms will likely operate in real time during intraoral scanning, offering immediate feedback on scan completeness, margin clarity, and potential errors [2,10]. Integration with next-generation scanning devices may allow AI to guide the clinician during acquisition, ensuring optimal data capture and reducing retakes, thereby improving the efficiency and accuracy of downstream design processes.

The application of AI in diagnostics continues to be developed. AI technologies in dentistry have the power to become central in the triad of patient data management, health care application, and services, and can facilitate future developments in patient-centered individualized treatment [29]. Despite the clear potential, several challenges must be addressed for widespread AI integration. Limitations include the need for large, high-quality datasets, challenges in standardizing data across different scanners and software systems, and concerns regarding privacy, ethics, liability, and clinician acceptance [1,8,9]. There is also a growing need for transparent and interpretable AI models to ensure that clinicians can understand system outputs and maintain responsibility for clinical decisions. Regulatory frameworks will need to adapt to AI-generated diagnosis and treatment plans, particularly when systems begin to operate with greater autonomy [7]. Education will also play a crucial role; incorporating AI training into dental curricula will be essential to prepare future clinicians for AI-enhanced workflows. Machine intelligence has the potential to be humanity’s final invention, representing a monumental breakthrough in history. However, it could also bring significant risks if we do not learn how to manage it responsibly [30].

In summary, the future of AI in dentistry, especially within digital dentistry, points toward increasing precision, automation, and personalization. Advances in GAN and CNN architectures, expansion of multimodal datasets, and the integration of robotics and predictive analytics will reshape how clinicians diagnose, plan, and deliver care. AI will not replace clinicians, but it will significantly augment their capabilities, reduce variability, and enhance efficiency across all phases of treatment. As these technologies evolve, they will contribute to a more standardized, reliable, and patient-centered digital dental ecosystem, ultimately improving clinical outcomes and reshaping the future landscape of restorative and prosthodontic practice [1-11,15,20,22-25].

Quality and Limitations of Included Evidence

The body of evidence reviewed is promising yet constrained by significant methodological heterogeneity. The majority of the literature consists of in vitro analyses, narrative reviews, or feasibility studies rather than large prospective clinical trials, which limits the direct translation of findings into routine clinical practice [5,8,20,22-26,29]. Furthermore, the lack of standardized reporting, evidenced by the use of diverse datasets, disparate software architectures, and varying evaluation metrics such as RMSD, Hausdorff distance, and IoU, complicates direct comparisons across studies and inhibits meta-analytical synthesis [20,23-26]. Additionally, many investigations are characterized by limited sample sizes and controlled laboratory settings, which may not fully reflect the complexities of patient-specific anatomical variations and clinical workflows [20,22-26]. While the available literature demonstrates a clear trend toward improved accuracy, consistency, and efficiency with AI-driven systems, further large-scale, longitudinal clinical studies are imperative to validate their long-term reliability and generalizability in diverse dental environments.

## Conclusions

The integration of AI into digital dentistry has transitioned from experimental theory to a clinically applicable reality, particularly within restorative and prosthodontic CAD workflows. Evidence demonstrates that AI-driven systems, specifically those utilizing GANs and CNNs, effectively address the limitations of conventional CAD by reducing operator dependency and enhancing design reproducibility. These technologies have shown superior precision in critical tasks such as marginal line identification, internal adaptation, and the generation of anatomically accurate crown morphologies. By leveraging DL from vast clinical datasets, AI models provide a level of consistency and predictability in restoration design that minimizes manual adjustments and improves patient-specific outcomes.

Ultimately, AI serves as a transformative supportive technology that enhances, rather than replaces, the clinician’s expertise and professional judgment. By standardizing digital workflows and improving the accuracy of occlusal and marginal fits, AI-based CAD systems allow for more efficient, patient-centered care and reduced chairside time. While ethical considerations and data quality remain essential for safe implementation, the demonstrated clinical benefits confirm that AI is a practical tool for modern restorative practice. Future integration will likely continue to refine these workflows, further bridging the gap between automated digital design and long-term clinical performance.
